# Buffalo Medical and Surgical Association

**Published:** 1883-01

**Authors:** Clayton M. Daniels

**Affiliations:** Secretary


### Society Reports.

### Buffalo Medical and Surgical Association.

*Regular Meeting, December 4th, 1882.*

Vice-President, Dr. John Cronyn, in the Chair. Dr. C. M. Daniels, Secretary.

Present—Drs. Bartlett, Phelps, Granger, Fowler, Tremaine, Brecht, Gay, Coakley, Hartwig, Briggs, Stockton, Wm. Ring, C. A. Ring, Macniel, McBeth, Hopkins, Johnson, and, by invitation, Drs. Clark, Waldurff, Hubbell, Frank, Ingraham, Peterson, Frederick and Long.

Applications for membership were received from Drs. Edward Clark and E. C. Waldurff.

Dr. Tremaine introduced the subject, "Strictures of the Urethra," and, in discussing it, spoke of their frequency, given by statistics as being 2600 in 150,000 cases of specific urethral diseases. Experience had taught him that there was even a greater proportion where large garrisons were located, gleet being something that was exceedingly familiar, and that there were various opinions regarding it. Anatomically speaking, the organ was of a complex nature, and there was a laxity regarding the cause or nature of strictures. The urethra, instead of being a tube and merely mechanical in character, is really a valve, being an outlet for semen, and, secondarily, for the urine. Also, the intimate connection between the urethra and the corpus spongiosum, together with the free intermingling of nerve fibres, made inflammatory action different in its result from that at other points. Stricture is never found in the prostatic portion, because the quantity of connective tissue is small, this portion of the urethra being of a musculo-glandular character.

The cause of stricture, nine times out of ten, is from urethritis, the product of inflammatory action, being a proliferation of connective tissue and neoplasm, destroying the dilatability of the canal, which gradually contracts, obstructing the flow of urine, and most frequently results in cystitis, to a greater or lesser degree. The tendency of the newly-formed connective tissue is to undergo a cartilaginous formation, and which is extremely slow in being absorbed. Many nervous symptoms are, at times, observed, as is great muscular debility, analogous to that in the female where cervicitis is present. Spasmodic stricture of the deeper portion of the urethra is referred to by Dr. Otis as being common and caused by reflex irritation only. The speaker considered this an extreme view, although agreeing that it was not unfrequently the case, and that such were sometimes mistaken for organic stricture.

The treatment varies according to location, the first effort being to try and pass the sound, bougie or whalebone probe, and over the whalebone probe, if used, a tunnel sound. If unable to do this, external urethrotomy is a last expedient. As to the advisability of cutting by internal urethrotomy, as advised by Dr. Otis, the speaker, from personal experience, had found it unsatisfactory in the deeper portions of the canal, although usually advisable when the stricture was within two inches of the meatus, eminent andrologists considering that in all other cases gradual dilatation is safest and best. As to the use of sounds, he thought that in general they were introduced too frequently, and that once in five to eight days was sufficient, giving time for the irritation to subside during the interval. Recommended the gum elastic bougie in diagnosing in preference to the bulbous metallic mass of Otis, which he thought might do harm. Advised over-distension where the calibre would admit, and recommended Gouley's instrument. As to the word *cure*, he thought it could not be applied to strictures, although practically such would sometimes seem to be the result and answer all purposes when the gleet disappeared and the obstruction was apparently removed. Regarding dilating bougies, up to No. 9 of the English scale the soft gum elastic are preferable, afterward the polished conical steel sounds, which seem to give even less pain. Said that some authorities say, "forget your anatomy when dealing with urethral strictures," but which to him would seem poor practice.

As to "electrolysis" he had no personal experience, although it had been highly recommended. Again, referring to the practice of cutting, he thought it was going out of fashion. Mentioned Dr. Eldredge, of Japan, who at one time endorsed but had now discontinued it, as had many others. Referred to the enlarged prostate as another form of obstruction, but without a discharge, and related Gouley's method for the removal of a part of the so-called "third lobe" with an instrument resembling the lithotrite, but with shorter jaw, which cut off the obstruction. It was rarely performed, and there is considerable controversy in England regarding it.

*Discussion*—Dr. Gay said he was exceedingly interested in the address of Dr. Tremaine, and thought that a few more papers of like ability would restore our association to its former prestige, when its papers were quoted and referred to all over the land. He had no criticisms to offer, and considered the teachings of Dr. Tremaine as sound, and especially regarding the urethra as a *valve* and an outlet, not an inlet. Pathologically, he had nothing to add. Considered the filiform bougie a great advance in urethral exploration, and if not irrelevant, he believed that a greater advance was in the demonstration of Dr. Otis, "that of the urethral calibre in relation to the flaccid penis." He thought that, possibly, older surgeons had not adopted the Otis idea; but, believing "a stricture once a stricture always," the speaker himself was one of that kind. He had treated many cases of stricture, and performed all operations for the same, and between division, dilatation, internal and external urethrotomy, agreed that we do as little cutting as possible. Sir Henry Thompson proposed that if No. 1 up to No. 6 be introduced, it then be divided; but, in opposition, Dr. Gay believed that if No. 6 could be used, we could gradually and certainly advance to No. 24.

Dr. Bartlett had not had extensive experience in the treatment of strictures, and asked to be informed as to the opinion of others in regard to the use of caustics, also regarding the length of time it was best to leave in the sound at each dilatation. His treatment of enlarged prostate had been quite successful where a soft catheter was left from twenty-four to forty-eight hours, which gave the needed relief, subsequent introduction being usually unnecessary. Thought that the ideas regarding internal urethrotomy were becoming more modified.

Dr. Phelps had been obliged to have considerable to do with strictures, especially in hospital practice, where the cases were usually extreme ones, and where previous unsuccessful attempts had been made to pass the catheter before the patient reached the hospital, which many times left the urethra in a very irritable condition, the passage being filled with blood clots, etc. Many times, when a case was urgent, had aspirated bladder by suprapubic puncture, and had never had any unfavorable results. Had found the filiform bougie invaluable, and explained his manner of introduction, saying that when one failed to pass it was left in situ, then another used, until the several false passages were filled, and, by persistent effort, he was always successful in reaching the bladder. Had used Gouley's divulsor, and recommended its use in the pendulous urethra; also, thought that slitting the meatus was often necessary for the introduction of a proper size sound.

Dr. Hartwig asked Dr. Tremaine to explain in what way his operations, as advised by Dr. Otis, had been unsatisfactory. He also strongly expressed his doubts as to Dr. Tremaine's statement regarding the catarrhal inflammation of the bladder following stricture, which he thought did not occur, and related cases where urine was retained for several weeks without such results. Said that Dr. Haney, of Germany, recommended that when the prostate was enlarged, it be injected through the rectum. The speaker had tried it, but with disastrous results.

Dr. Cronyn was sorry that the time given to discuss the subject was so short, and thought it might be well to continue it at a future meeting, as it was certainly one of great importance. He thought where any instrument, however small, could be passed into the bladder, the word "stricture" did not apply; for, as Syme says, "no stricture is impassable that anything can be passed through." He had not adopted the whalebone bougie, not yet having found it necessary. Thought that the failure in passing a catheter was sometimes due, especially in young practitioners, to a lack of knowledge as to the anatomy of the parts. If an anæsthetic was necessary, advised using as large a catheter or sound as possible, it being less dangerous. Spoke of the various instruments, as one invented by Wakely, of London; also, that of Sir Henry Thompson, which is very heavy and easily passes the ordinary stricture, and which is highly praised by many, especially Hutchinson, of Brooklyn. The speaker had used it often, and for all passable strictures it was the most ready and satisfactory. Thought that stricture in the prostatic portion of the urethra only occurred after an abscess or similar cause. Referring to the many instruments invented, and so highly lauded by their inventors, he said that doubtless each could use his own best, and that, in general, in the hands of others they were far less satisfactory. He considered the best and surest operation for organic stricture is external perinæal urethrotomy; cutting on the inside he opposed, as more accidents are liable to follow, such as urethral fever, pyæmia, etc. Related some personal experiences which confirmed his views. The question of caustics was, to his mind, now out of date.

Dr. Tremaine, in conclusion, referred to the urethral irritation that sometimes occurs, followed by a constant discharge, and independent of any specific cause, which gives rise to no little alarm, but which, however, is non-infecting, and can usually be successfully treated by irrigating the urethra daily, using a return current catheter and a solution of borax, glycerine and water; although he has, in extreme cases, found it necessary to use a solution of nitrate of silver, five grains to the ounce of water. Regarding Dr. Phelps' procedure in tapping the bladder by supra-pubic puncture, he would not advise it unless immediate relief was absolutely necessary and other measures not at hand. In due deference to Dr. Hartwig, he felt bound to affirm that prolonged stricture *always* produced cystitis. Also, in reply, regarding the result of some of his operations by Otis' method, they were nearly all negative, and of eight cases no material benefit was obtained. Thought that the operation of external urethrotomy was a delicate one, even under favorable circumstances, and especially if attempted without a guide. Various instruments were exhibited.

Dr. Cronyn, in explaining the evident difference of opinion between Drs. Tremaine and Hartwig regarding cystitis, said that in the cases related by Dr. Hartwig there was no evidence of purulent contamination of the urine in the bladder, as would be the case in stricture from some specific cause; and that it was well known that urine, of itself, in the bladder, would not cause cystitis.

Adjourned.

CLAYTON M. DANIELS, *Secretary.*

